# In Silico Drug Repurposing in Multiple Sclerosis Using scRNA-Seq Data

**DOI:** 10.3390/ijms24020985

**Published:** 2023-01-04

**Authors:** Andrey Shevtsov, Mikhail Raevskiy, Alexey Stupnikov, Yulia Medvedeva

**Affiliations:** 1 Institute of Bioengineering, Research Center of Biotechnology, Russian Academy of Science, 117312 Moscow, Russia; 2 École Polytechnique Fédérale de Lausanne (EPFL), 1015 Lausanne, Switzerland; 3 I.M. Sechenov First Moscow State Medical University, 119991 Moscow, Russia; 4 Department of Biomedical Physics, Moscow Institute of Physics and Technology, 141701 Dolgoprudny, Russia; 5 The National Medical Research Center for Endocrinology, 117036 Moscow, Russia

**Keywords:** multiple sclerosis, drug repositioning, scRNA sequencing, connectivity mapping, LINCS L1000

## Abstract

Multiple sclerosis (MS) is an autoimmune disease of the central nervous system still lacking a cure. Treatment typically focuses on slowing the progression and managing MS symptoms. Single-cell transcriptomics allows the investigation of the immune system—the key player in MS onset and development—in great detail increasing our understanding of MS mechanisms and stimulating the discovery of the targets for potential therapies. Still, de novo drug development takes decades; however, this can be reduced by drug repositioning. A promising approach is to select potential drugs based on activated or inhibited genes and pathways. In this study, we explored the public single-cell RNA data from an experiment with six patients on single-cell RNA peripheral blood mononuclear cells (PBMC) and cerebrospinal fluid cells (CSF) of patients with MS and idiopathic intracranial hypertension. We demonstrate that AIM2 inflammasome, SMAD2/3 signaling, and complement activation pathways are activated in MS in different CSF and PBMC immune cells. Using genes from top-activated pathways, we detected several promising small molecules to reverse MS immune cells’ transcriptomic signatures, including AG14361, FGIN-1-27, CA-074, ARP 101, Flunisolide, and JAK3 Inhibitor VI. Among these molecules, we also detected an FDA-approved MS drug Mitoxantrone, supporting the reliability of our approach.

## 1. Introduction

Multiple sclerosis (MS) is a common autoimmune-mediated neurodegenerative disease of the central nervous system (CNS). A characteristic feature of this disease is inflammatory demyelination with axonal transection. MS typically appears in young adults (20–30 years) and can lead to physical disability, cognitive impairment, and decreased quality of life [1]. The first clinical onset of MS is called clinically isolated syndrome (CIS). The disease is traditionally divided into three types: a relapsing-remitting multiple sclerosis (RRMS) with attacks of new or increasing neurologic symptoms followed by periods of partial or complete recovery, a secondary progressive MS (SPMS) with a non-relapsing progression followed by RRMS, and a primary progressive multiple sclerosis (PPMS) with initial non- relapsing progression [2,3]. 

Since an autoimmune process in CNS causes MS, it is usually explored using either peripheral blood mononuclear cells (PBMC) or cerebral spinal fluid (CSF). The inflammatory infiltrates contain T-lymphocytes, dominated by MHC class I restricted CD8+ T-cells; B cells and plasma cells are also present, although in much lower numbers [4]. CSF is usually used for MS diagnostics, while PBMC are frequently used for investigating MS. For MS patients, a specific group of proteins called oligoclonal bands (bands of immunoglobulins) is usually detected [5].

There is no cure for multiple sclerosis. Treatment typically focuses on speeding recovery from attacks, slowing the progression of the disease, and managing MS symptoms. The first drug for MS was Interferon beta-1b (IFN-ß), approved in the 1990s for RRMS to modulate the progression of the disease. The effects of IFN-ß are complex and have not been explored in detail. IFN-ß binds to the type I IFN receptors, with higher affinity to INFAR-2 than to INFAR-1. This binding activates the JAK/STAT signaling pathway regulating multiple key cytokines, by which JAK/STAT regulates various T-cell subpopulations [6]. Over the past two decades, treatment strategies have changed dramatically. Currently, more than a dozen drugs have been created and approved for RRMS, and one agent has been approved for PPMS [7]. RRMS-targeting agents include interferon beta, glatiramer acetate, fingolimod, siponimod, mitoxantrone, teriflunomide, dimethyl fumarate, cladribine, alemtuzumab, ocrelizumab, and natalizumab. The PPMS-targeting drug is ocrelizumab. These agents represent multiple substance classes with different modes of action [8]. Fingolimod is used for the retention of auto-reactive lymphocytes within secondary lymphoid organs. It is an antagonist of S1PR1, essential for lymphocytes to egress from secondary lymphoid organs into the systemic circulation [9,10]. Natalizumab, similarly to fingolimod, blocks the transporting activities of immune cells. It blocks the α4 subunit of integrin and thus inhibits leukocyte extravasation into the CNS and intestinal tract [11]. Mitoxantrone is used for its immunosuppressive effect on proliferating B and T lymphocytes via induction of cell lysis and initiation of programmed cell death [12,13]. Teriflunomide inhibits the mitochondrial enzyme dihydroorotate dehydrogenase (DHODH), which reduces the proliferation of activated T and B cells. Dimethyl fumarate activates the Nrf-2 pathway, which leads to an expansion of FoxP3+ regulatory T cells and CD56 bright natural killer cells, as well as to a reduced level of CD8+ T cells and B cells [14]. Cladribine is a prodrug; after activation, it induces cell death. Alemtuzumab is an antibody that targets CD52 T and B cells. For now, it is clear that B cell function is critical for MS. Many drugs to reduce the number of immature and mature B cells in MS have been developed, including ocrelizumab, an antibody against CD20 B cells. Despite significant improvement over the last few decades, currently used drugs still have severe side effects and do not work in many cases, which makes the search for new drugs against this disease an actual and important problem.

The diversity of cell types involved in the onset and progression of MS makes single-cell RNA sequencing (scRNA-seq) one of the most promising methods to investigate the pathways affected in MS. scRNA-seq makes it possible to detect changes within specific cell types in MS seeking to control the effect of the cell proportions change. Additionally, having clear transcription signatures of a particular cell type in the MS sample and discriminating it from the same cell type in a non-MS sample, provides one with a valuable source for drug repositioning. One of the approaches to searching for drugs or small molecules using transcriptional signatures is connectivity mapping (CMap). CMap is a method to find a drug with a given transcriptional effect. CMap is based on the assumption that a set of important up- and down-regulated genes (a transcriptional signature) should be similar for similar drugs or states. Such signatures reflect the difference in the expression profiles of the cells of the two compared states. When we know the expression signature determining the difference in phenotypes of the compared cohorts, we can compare it with the expression signatures of the chemical agents. The similarity is calculated based on the value of an input metric that takes into account the proximity of the lists of regulated genes and the direction of regulation. Thus, it is possible to compile a list of chemical compounds from the database that can potentially change the expression profile of a cell (transfer from one state to another) [15]. LINCS L1000 [16] is currently the most popular database containing transcriptional signatures of different drugs. 

In this study, we explored the public data of peripheral blood mononuclear cells (PBMC) and сerebrospinal fluid (CSF) cells of patients with MS and idiopathic intracranial hypertension (IIH). We demonstrate that AIM2 inflammasome (in pDCs and mDCs), SMAD2/3 (in T cells, granulocytes, and mDCs) signaling and complement (classical and alternative) activation pathways are affected in MS. Using genes from top-activated pathways as transcriptional signatures for the Cmap approach, we detected several potentially effective small molecules that might compensate for the transcriptional changes in cells of MS patients, including 16-hydroxytriptolid, AG14361, FGIN-1-27, CA-074, ARP 101, geldanamycin, Flunisolide, PX12, and JAK3 Inhibitor VI for granulocytes. Several of these molecules have already been tested on mouse MS models (EAE), e.g., FGIN-1-27, CA-074, and mTOR inhibitors. Among these molecules, we also detected an FDA-approved MS drug Mitoxantrone, validating our approach.

## 2. Results and Discussion

### 2.1. Changes in Pathway Activation

For this study, we used scRNA-seq data from PBMS and CSF from patients with MS and a control cohort of patients with idiopathic intracranial hypertension [17].

**Figure 1:**
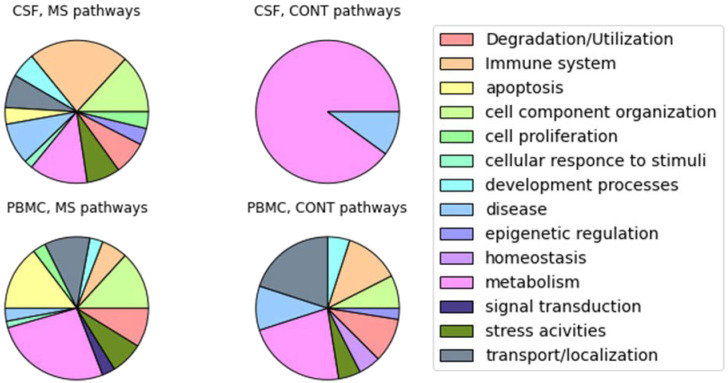
A summary of the unique pathway categories in MS state compared to control (CONT) detected for each cell type separately. For a better understanding of the overall picture of the processes, pathways are grouped into generalized categories.

Using the OncoboxPD [18], we detected activated and inhibited pathways in the CSF and PBMC of MS patients (Figure 1). Unique pathways were grouped into categories based on integrating categories from multiple databases (Bicarta, Qiagen, Reactome, NCI, KEGG). For CSF cells in MS patients, pathways related to the immune system were the most frequently activated (intestine IgA production, downstream signaling in naive CD8 T cells, regulation of survival gene product expression via IL2RG, TCR signaling in naive CD8 T cells (regulation of survival gene product expression via AKT1), antigen presentation folding assembly and peptide loading of class I MHC, classical antibody-mediated complement activation, endosomal vacuolar pathway, FCGR activation, initial triggering of complement, PD 1 signaling, phosphorylation of CD3 and TCR zeta chains, phospholipids in phagocytosis, translocation of ZAP 70 to Immunological synapse), which was expected for MS. For PBMC, the majority of activated pathways were related to metabolism, cell component organization, and apoptosis. The latter plays an important role in the immune regulation of MS via activation-induced T-cell death and local processes of tissue damage [19].

Following Schafflick and colleagues [17], we clustered the cells in each dataset (disease and control samples) separately and determined cell types in CSF and PBMC using markers provided in Appendix A Table A1. When investigating pathways activated in particular cell types, we found specific ones directly linked to MS (Supplementary Tables S1 and S2). For example, in PBMC, the AIM2 inflammasome-associated pathway is activated in pDCs and mDCs. AIM2 inflammasome represents a structure detected in MS that can be used as a therapeutic target [20]. Regulation of survival gene product expression via the IL2RG pathway is activated in CSF mDCs. Although IL2RA has been reported as a risk factor for MS [21], IL2RG has not been recognized as directly linked to MS. Still, IL2RG might be involved in general autoimmune processes. 

One of the major conclusions of Schafflick and colleagues [17] was that, in MS patients, diversity in cell types and expression increased in CSF and PBMC, respectively. In line with this observation, we report the tremendously increased diversity of activated pathways in the CSF of MS patients (Figure 1).

Interestingly, in control PBMC, immune pathways connected to inflammation were activated. The TRAF6-mediated induction of the proinflammatory cytokines pathway was found activated in multiple cell types (gd T cells, granulocytes, naïve B cells). TRAF6 participates in protective responses in immune and non-immune cells [22], suggesting that control samples may also reflect inflammation typical for hypertension (reviewed in [23]). We also found complement activation pathways. It is of note that in CSF (plasmablasts) a classic complement pathway has been activated, while in PBMC (granulocytes, monocytes) an alternative complement pathway has been activated. Although, in the article, both pathways are mentioned as important for MS development on mouse models [24]. Additionally, multiple VEGF-connected pathways are activated in CSF (activated CD8+ T cells, gd T cells, ab T cells, monocytes) and PBMC (mDCs and megakaryocytes) of MS. VEGF is a neuroprotective agent [25] and was shown to be increased in MS [26].

### 2.2. Small Molecules Potentially Effective against MS

**Figure 2:**
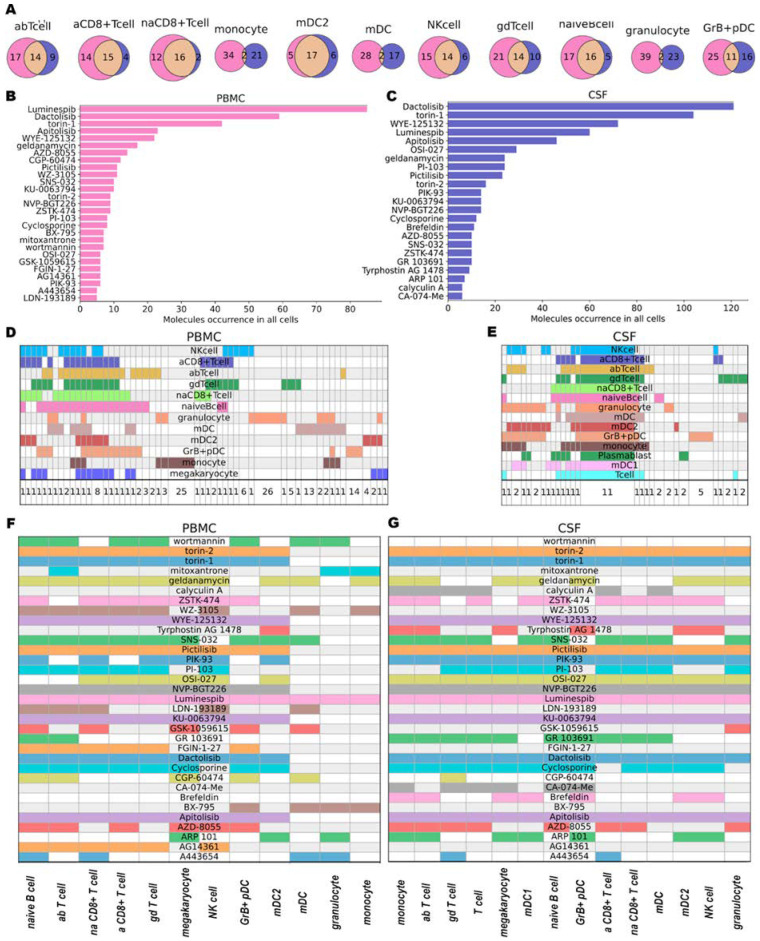
Small molecules results (**A**): Intersections of the same cell types in PBMC (purple) and CSF (blue). (**B**,**C**): Occurrence of molecules in CSF (**C**) and PBMC (**B**) in all cell types. For visualization, we used only molecules that occur more than five times. (**D**,**E**): Intersection of agents obtained for each cell type in tissue (PBMC—(**D**), CSF—(**E**). Each column represents a group of small molecules (their number is provided at the bottom of the plot). Groups were created and sorted based on intersections of small molecules between cell types (rows). (**F**,**G**): Plots of the occurrence of molecules in the found cell types in PBMC (**F**) and CSF (**G**).

Encouraged by the fact that by using scRNA-seq data it was possible to detect distinct pathways related to MS activated in different cell types, we sought drugs potentially capable of reverting the transcriptional signature of cells affected by MS using a CMap approach (see Methods). Using differentially expressed genes in each cell type we obtained the top 50 molecules in PBMC and CSF with the highest likelihood to reverse changes observed in MS. In total we acquired 155 and 50 unique molecules for all cell types in PBMC and CSF, respectively. For the majority of cell types, we have a significant intersection of predicted drugs between various sources of cells (Figure 2A). Only for monocytes, mDCs, and granulocytes, were the intersections small, presumably due to variability of the transcription profile of these cells in different sources. Both PBMC and CSF have the same top five most-frequent molecules (Luminespib, Dactolisib, Torin-1, Apitolisib, and WYE-125132) (Figure 2B,C). These may indicate that, despite differences at transcriptional levels and cell type content in CSF and PBMC, both cell types have common activated pathways. For CSF, 11 molecules (Luminespib, Apitolisib, Pictilisib, KU-0063794, Dactolisib, NVP-BGT226, OSI-027, PIK-93, WYE-125132, Torin-1, Torin-2) were common to all cell types (Figure 2E), while only one molecule (Luminespib) was also common to all cell types in PBMC (Figure 2D). Among the frequent molecules, (Figure 2B) we also found Mitoxantrone—an FDA-approved drug used for some MS patients—supporting the reliability of our approach. All found molecules are presented in Supplementary tables (Tables S3 and S4).

#### 2.2.1. Molecules Affecting Multiple Cell Types

More than half of the most frequently found molecules in both CSF and PBMC (Figure 2F,G) were mTOR and PIK3 inhibitors (Dactolisib, Torin-1, WYE-125132, Apitolisib, OSI-027, Pictilisib, PI-103, Torin-2, PIK-93, KU-0063794, NVP-BGT226, ZSTK-474, AZD-8055, wortmannin, GSK-1059615). mTOR plays an important role in the signaling network that regulates growth and metabolism in response to environmental cues [27]. Multiple experiments on special mouse models for MS (Experimental autoimmune encephalomyelitis (EAE)) (reviewed in [28]) support the hypothesis that the mTOR network may have significant involvement in the pathogenesis of MS. Downregulation of the mTOR leads to the reduction of MS inflammatory processes in EAE models [29,30,31,32]. mTOR inhibition in B cells leads to a dual immune effect. mTOR supports the proliferation/clonal expansion of B cells and plasma cells and many differentiated T cell subtypes, as well as the differentiation of B cells [33]. One of the mTOR inhibition consequences is autophagy activation, Tregs increase, T cell anergy, and T-effector decline. On the one hand, autophagy leads to inflammation reduction via inflammasome inactivation; on the other hand, autophagy inducers could also promote autoimmunity [34]. Moreover, mTOR inhibition promotes proinflammatory M1 macrophage polarization. Also, mTOR inhibition in human isolated monocytes and mDC activates the production of proinflammatory cytokines (IL-12, IL-23, IL-6, and tumor necrosis factor α, TNFα) while it inhibits the release of the anti-inflammatory cytokine IL-10 [35]. These opposite effects suggest that it could be beneficial to activate mTOR in some cells (DC or B cells) and inhibit it in others (T cells) [36]. We detected mTOR/PIK3L/AKT inhibitors in every cell type in both BPMC and CSF, making mTOR a promising target for MS therapy. 

Among other frequently detected small molecules, we found the HSP90 inhibitors Luminespib and Geldamycin (Figure 2F,G). Luminespib was predicted in every cell type in both PBMC and CSF. Geldanamycin was ranked 7th in CSF and 6th in PBMC (Figure 2B). A cellular chaperone, HSP90 is important for the folding and function of protein kinases, steroid hormone receptors, and various cellular proteins and also regulates the cell cycle and apoptosis [37]. Inhibition of HSP90 leads to activation of the Heat Shock Response, which can reduce the incidence and severity of MS, as tested on EAE models [38]. It also causes the activation of cytoprotective chaperones, which are involved in neuroprotective processes against the deficiency of Purkinje cells [39]. Despite the promising effects of HSP90 inhibitors, they also demonstrate high cytotoxicity. Luminespib was used in clinical trials against metastatic pancreatic adenocarcinoma but did not complete phase II clinical trials [40]. Geldanamycin successfully passed the first clinical trials [41] but also demonstrated cytotoxicity reducing researchers’ interest in this molecule. Recently, Geldanamycin has been explored as an effector on HSP90 in neurodegenerative diseases [42]. 

In addition, we detected several immunosuppressors, mostly T cells activity suppressors, including Cyclosporine, ARP 101 (Figure 2F,G), and less frequently 16-hydroxytriptolide. Unfortunately, Cyclosporine demonstrated significant toxicity in effective doses [43]. Autophagy activation caused by ARP has a dual effect on inflammation in different cells [44]. Although, ARP 101 showed a potential effect on Sjögren’s Syndrome (autoimmune, inflammatory disease) [45]. 

Among the drugs predicted for multiple cell types in CSF molecules was GR 103691, a dopamine D3 receptor antagonist (Figure 2G). Dopamine modulates Th17 cell function as well as dendritic cell-mediated Th17-immune response—a critical player in the pathogenesis of multiple sclerosis—suggesting that dopaminergic receptors could serve as new therapeutic targets in MS (reviewed in [46]). 

Cathepsin B Inhibitor (CA-074) was found only in CSF but for multiple cell types (Figure 2G). Cathepsin B plays an important role in physiological and pathological neuronal processes. CA-074 forces anti-inflammatory processes on the EAE model by decreasing the number of Th1, Th17, and Th22, and increasing the number of Tregs [47]. 

An anxiolytic drug, FGIN-1-27, has been detected in multiple cell types (mostly in T cell subtypes) of PBMC (Figure 2F). FGIN-1-27 affects the production of IL-17 and reduces the pathogenicity of Th17 cells in the mouse EAE model, making it a potential agent against MS [48]. 

AG14361 (PARP1 inhibitor) has been detected for all our PBMC subtypes of T cells as well as in NK cells and naive B cells (Figure 2F). Poly (ADP-ribose) polymerase 1 (PARP-1) is essential in immune and inflammatory responses. PARP1 was shown to mediate necrotic neuron death or to protect neurons in different cases [49]. It is still unclear if PARP1 should be activated or inhibited to reduce symptoms of MS. However, PARP1 expression is reported to be higher in CD4+ and CD8+ cells in MS [50], suggesting that PARP1 inhibitors could be beneficial in MS.

#### 2.2.2. Molecules Affecting Particular Cell Types

PX12—an inhibitor of Trx-1 (thioredoxin-1)—has been detected for granulocytes and NK cells in PBMC. It stimulates apoptosis, downregulates HIF-1α and vascular endothelial growth factor (VEGF), and inhibits tumor growth in animal models [51]. It is a bit controversial to our pathways analysis since the VEGF pathway was found in PBMC for mDC and megakaryocytes, and in CSF for multiple T cells, and NK cells. The reason for this may lie in the large number of diverse functions of thioredoxin-1, so, for these cells, PX12 was chosen based on other pathways.

Interestingly, for monocytes in PBMC, we found the anti-asthma drug Flunisolide, which is a corticosteroid effective for controlling chronic lung inflammatory diseases [52,53]. We did not find any experimental evidence of Flunisolide effects in the MS state.

Additionally, JAK3 Inhibitor VI has been detected among molecules affecting granulocytes in PBMC. JAK/STAT activation by proinflammatory cytokines has a great impact on the pathogenesis of MS [54]. JAK inhibitors are being explored for use in different autoimmune diseases [52,55].

## 3. Materials and Methods

### 3.1. Data

We used scRNA-seq data from a single experiment [17]. The dataset contained transcriptomes of individual CSF cells (six MS patients and six with idiopathic intracranial hypertension as a control) and PBMC (five MS patients and five patients with idiopathic intracranial hypertension). The cohort of MS patients contained both CIS and RRMS.

### 3.2. Processing of Single Cell RNA Seq Data

We used the R programming language (version 4.1.1) and Seurat package v.4.1 [56]. We filtered raw count data by the number of genes and cells (min.features = 200, max.features = 2500, min.cells = 3), by the percentage of mitochondrial (<10%), hemoglobin (<10%) genes. We normalized the data using a regularized binomial regression model (SCTransform function with default parameters). Then we integrated the data from all donors. We reduced the dimension of the integrated data by PCA (30 dims). For visualization, we used the UMAP (runUMAP function) method, based on 30 PCA components. We performed clustering of the obtained embeddings based on the PhenoGraph method [57], for this purpose, neighbors were first determined by the Findneighbours function, and then clusters were determined by the FindClusters function (resolution = 0.5). We determined cell types based on markers for each of the obtained clusters. To do this, we found marker genes for each cluster (log2FC = 0.25, p-val = 0.01, min.pct = 0.3). For the annotation, we searched for markers of the most common immune cell types. We took marker genes from the article with the analyzed data as a basis. All used markers are in Table A1.

### 3.3. Determination of Activated Biological Pathways

Activation/inhibition of biological pathways was determined by geometric mean for all genes in the pathway for all cells annotated as one cell type. Activated pathways were determined using the OncoboxPD [18], in which the activation level of the regulatory pathway (PAL) is calculated for each biological pathway from six databases (Biocarta [58], Reactome [59], KEGG [60], Qiagen Pathway Central [61], NCI [62] and HumanCYC [63]) based on expressed genes. Ten pathways with the highest PAL were selected as the main processes taking place in cells.

### 3.4. Determination of Small Molecules

Small molecules potentially capable of changing the expression profile of fibroblasts into cardiomyocytes were identified using the L1000CDS service (https://maayanlab.cloud/L1000CDS2, accessed on 6 December 2022), based on the principle of connectivity mapping and the L1000 database. Transcriptional signatures from the database are ranked based on interception query upregulated and downregulated genes. One molecule can occur multiple times if multiple experiments with different dosages or cell types had a significant gene intersection. In our research, for each cell type, we formed two lists with genes. The first list contains genes from the top 10 upregulated pathways. The second list contains genes from 10 downregulated pathways. We used the L1000CDS2 API to make queries. We did not choose special cell types to search for all cell types in the database. We chose reverse mode (parameter “aggravate” = False) to find molecules that can inhibit MS-activated pathways and activate non-MS-activated pathways. Genes from the top 10 activated and inhibited pathways were used for a small molecules search. We obtained small molecules for each cell type separately.

## 4. Conclusions

In this study, we computationally screened for pathways affected in MS for different cell types and drug candidates against MS using scRNA-seq data and the CMap approach. We analyzed expression data in the PBMC and CSF of MS patients compared to the control group with idiopathic intracranial hypertension. Based on genes from top-activated pathways we found small molecules with the potential to reverse the MS state. Analysis of the MS state in all cell types separately allowed us to find agents to target exact cell types independently (e.g., PX12 for granulocytes and NK cells in PBMC or Flunisolide for PBMC monocytes) as well as to detect molecules that can affect multiple cell types (e.g., mTOR inhibitors). We detected the connections between obtained biological pathways and mechanisms of action of detected small molecules. Inflammasome activation can be reduced by activation of autophagy, so we found mTOR inhibitors and also ARP 101, whose consequence is autophagy activation. Proinflammatory processes and complement activation are the signals of an immune response, so detection of immunosuppressors (Cyclosporine, ARP 101, 16-hydroxytriptolide) and anti-inflammatory agents (CA-074, Flunisolide, JAK3 Inhibitor VI) supports the reliability of our approach. We also found VEGF pathways upregulated in MS state and VEGF inhibitors among potentially active small molecules but, surprisingly, in different cell types. The pathway for CSF is activated CD8+ T cells, gd T cells, ab T cells, monocytes, and, for PBMC, in mDCs and megakaryocytes. Small molecules were found for granulocytes and NK cells in PBMC. 

Schafflick and colleagues hypothesized that follicular T cells were involved in B cell promotion [17]. These findings stress the importance of a search for the agents targeting this helper T cell subpopulation. In our research, we did not manage to detect CD4+ T cells and hypothesized that these cells were included in the T cell cluster population with other unclassified T cell types. This is probably the reason why we did not observe agents targeting exactly follicular T cells but some other helper T cells (e.g., CA-074). Molecules to affect B cells in CSF were mostly mTOR inhibitors, that inhibit B cell promotion, which also conforms with the B cell expansion processes during MS.

Based on our results and additional literature search, we selected the most promising molecules that we can suggest for further research: AG14361, FGIN-1-27, CA-074, ARP 101, PX12 for granulocytes and NK cells, and Flunisolide and JAK3 Inhibitor VI for granulocytes. Additionally, we think those mTOR inhibitors should also be deeply explored. Our research shows that mTOR inhibitors are effective for all observed cell types but this should perhaps be tested for the exact inhibition of mTOR in different combinations of immune cells to reduce side effects.

Cmap has great potential for drug repurposing but also has limitations. Though the approach may suffer from a high false positive rate and poor reproducibility [64], it allows a reduction of the number of promising molecules without huge biology experimentation on cell models for each molecule. There are more than 200,000 signatures in the L1000 database that cover more than 15,000 chemicals to obtain fast and flexible searches [65]. 

One of the key features of MS is the diversity of symptoms and disease triggers between patients [66]. This is a limitation of our approach since we merged data from only six patients, which most likely do not cover the whole spectrum of MS cases. At the same time, this diversity creates great opportunities for personalized approaches. With the increased sequencing depth, our approach to drug repositioning is potentially capable of providing personalized treatment suggestions.

## Data Availability

Code for the analysis is available at https://github.com/Barabaika/Single_cell_csf (accessed on 6 December 2022).

## Supplementary Materials

The supporting information can be downloaded at: https://www.mdpi.com/article/10.3390/ijms24020985/s1.

## Appendix A

**Table A1:** Cell types defining gene markers.

**Cell Type**	**Markers**
ab T cell	CD3E, LCK, TRAC
CD4+ T cell	IL7R, CD4
a CD8+ T cell	CD8B, CCL5, CD8A
na CD8+ T cell	CD8na, CCR7
Treg cell	FOXP3, CTLA4
gd T cell	TRDC
Th2 cell	GATA3, CCR3, CCR4
T cell	CD7
a T cells, NK, Th1	CXCR3
cytotoxic T/NK cell	GZMB
NK cell	GNLY, NKG7
NK1 cell	PRF1
NK2 cell	SELL, XCL1, CD62L
B cell	CD79A
naive B cell	IGHD, CD37
a B cell	CD27, IGHM
Plasmablast	IGHG, CD38, TNFRSF17, CD269
mDC	LYZ
mDC1	WDFY4, XCR1, BATF3
mDC2	FCER1A, CD1C, CLEC10A
pDC	TCF4, E2-2, TNFRSF21, DR6
monocyte	FCGR3A, CD16, CD14
granulocyte	S100A8, S100A9
megakaryocytes	GNG11, CLU
macrophages/monocytes	CD68
